# Exploring LLM-Based Generative Recommender Systems: Corpora, Customization, and Evaluation Insights

**DOI:** 10.1093/geroni/igaf122.2248

**Published:** 2025-12-31

**Authors:** Shuqi Yang, Mingrui Jing, Shuai Wang, Jiaqing Wang, Weijie Xing, Yan Hu, Zheng Zhu

**Affiliations:** Fudan University, Shanghai, Shanghai, China (People’s Republic); Fudan University, Shanghai, Shanghai, China (People’s Republic); Dali University, Dali, Shanghai, China (People’s Republic); Fudan University, Shanghai, Shanghai, China (People’s Republic); Fudan University, Shanghai, Shanghai, China (People’s Republic); Fudan University, Shanghai, Shanghai, China (People’s Republic); Fudan University, Shanghai, Shanghai, China (People’s Republic)

## Abstract

Large Language Model-Driven Generative Recommender Systems (LLM-GRSs) are increasingly transforming healthcare, particularly in question-answering systems. This study systematically reviewed their corpora sources, customization techniques, and evaluation metrics. A search of PubMed/MEDLINE, Embase, Scopus, and Web of Science identified 61 studies (2021–2024) using LLM-GRSs for medical information delivery. Corpus sources were categorized into real-world clinical resources (n = 24), literature materials (n = 34), open-source datasets (n = 33), and web-crawled data (n = 11), with 44 studies integrating multiple sources. Key model customization strategies included pre-training, prompt engineering, retrieval-augmented generation (RAG), fine-tuning, in-context learning, and offline learning. Fourteen studies used a single customization technique, while 41 studies combined these methods during model development. The evaluation metrics were classified into three main domains: 1) process metrics, 2) usability metrics, and 3) outcome metrics. The outcome metrics could also be divided into two categories: model-based outcomes and expert-assessed outcomes. The study identified critical gaps in corpus fairness, contributing to biases from geographic, cultural, and socio-economic factors. The reliance on unverified or unstructured data highlights the need for better integration of evidence-based clinical guidelines. Future research should focus on developing a tiered corpus architecture with vetted sources and dynamic weighting, while ensuring model transparency. Additionally, the lack of standardized evaluation frameworks for domain-specific models calls for comprehensive validation of LLM-GRSs in real-world healthcare settings.

